# Similarity of patient characteristics and outcomes in consecutive data collection on stroke admissions over one month compared to longer periods

**DOI:** 10.1186/1756-0500-7-342

**Published:** 2014-06-06

**Authors:** Chun Shing Kwok, Stanley D Musgrave, Gill M Price, Genevieve Dalton, Phyo Kyaw Myint

**Affiliations:** 1 Faculty of Medicine & Health Sciences, Norwich Medical School, Norwich Research Park, Norwich, UK; 2 Stroke Research Group, Acute Stroke Unit, Norfolk and Norwich University Hospital, Colney Lane, NR5 7UY Norwich, UK; 3 Institute of Cardiovascular Sciences, University of Manchester, Manchester Royal Infirmary, Manchester, UK; 4 School of Medicine & Dentistry, University of Aberdeen, Aberdeen, Scotland, UK; 5 Anglia Stroke & Heart Clinical Network, Cambridge, UK

**Keywords:** Stroke, Methodology, Data collection instruments, Mortality

## Abstract

**Background:**

The usefulness of time-limited consecutive data collection compared to continuous consecutive data collection in conditions which show seasonal variations is unclear. The objective of this study is to assess whether one month of admission data can be representative of data collected over two years in the same hospitals.

**Methods:**

We compared the baseline characteristics and discharge outcomes of stroke patients admitted in the first month (October 2009) of the Anglia Stroke Clinical Network Evaluation Study (ASCNES) with the routinely collected data over 2 years between September 2008 and April 2011 from the same 8 hospital trusts in the Anglia Stroke & Heart Clinical Network (AS&HCN) as well as seasonal cohorts from the same period.

**Results:**

We included a total of 8715 stroke patients (October 2009 cohort of ASCNES (n = 308), full AS&HCN cohort (n = 8407 excluding October 2009)) as well as cohorts from different seasons. All cohorts had a similar median age. No significant differences were observed for pre-stroke residence, pre-stroke modified Rankin, weekend vs. weekday admission, time of admission, patients with atrial fibrillation, type of stroke, admission systolic blood pressure, use of thrombolysis (rTPA), in-patient mortality and discharge destination. There were statistically significant differences between cohorts with regard to Oxfordshire Community Stroke Project Classification.

**Conclusions:**

Stroke patients admitted in one month had largely indistinguishable characteristics and discharge outcomes to those admitted to the same trusts in three separate seasons and also over two years in this cohort.

## Background

Study methodology is paramount because it affects the quality of the data, hence the interpretation of results and future clinical implications. The duration of the study is an important variable which in stroke studies can be influenced by case-mix and seasonal variations. Case-mix, the array of different disease and health problems treated [1], (in this case, stroke severity and age structure of patients etc.) influences hospitals’ performance which is of relevance to patients, clinicians, commissioners and service providers. For research studies such as clinical trials, case-mix match between intervention and controls is important so that results are due to interventions studied and not due to observed differences in case-mix.

Keeping the duration of data collection to a minimum is of interest because collecting data is time consuming, expensive and resource draining. The duration of data collection has relevance in conducting clinical audits where there are clinical implications for conducting audit cycles in a timely manner. Changes implemented after an initial audit should be evaluated quickly in order to determine if the changes should be abandoned or continued. In addition, understanding the minimum data collection period is useful in the context of pilot studies because there may be cases where the study duration of pilots may be affected based on the understanding of minimum data collection periods. This is of particular importance in conditions such as stroke where there is seasonal variation in incidence and outcome.

Several multicenter audits and registry studies have evaluated outcomes in stroke using different data collection methods and study durations. The UK National Stroke Sentinel audits collected data for three month periods for three different years with collection of a median of 40 patients from each site [2]. The Scottish Stroke Care Audit collects a core data set for each episode that has led a patient to be referred to hospital [3]. The New Zealand National Acute Stroke Services Audit 2009 reviewed up to 40 stroke patient records from 21 health boards over five months [4]. The China National Stroke Registry recruited 21,902 patients over 12 months from 132 hospitals [5]. The German Stroke Registers Study Group evaluated stroke care during a 2-month period in 13 hospitals [6]. The Get With the Guidelines-Stroke program has been used to evaluate secondary prevention measures [7] and validate data quality [8]. However, the minimum data collection period and minimum number of consecutive admissions with stroke to allow for adequate case-mix in evaluating outcomes has not been examined previously.

The objective of this study is to assess whether one month of admission data collected from eight NHS hospital trusts in the Anglia Stroke Clinical Network Evaluation Study (ASCNES) as a minimum data collection period is representative of data collected over two years covering all seasons by the Anglia Stroke & Heart Clinical Network (AS&HCN) in the same hospitals.

## Methods

### Study description

The Anglia Stroke Clinical Network Evaluation Study (ASCNES) utilises routinely collected data from a subset of the patients in the Anglia Stroke & Heart Clinical Network (AS&HCN) or ‘full cohort’ in order to maximise the benefit with minimal investment to produce the best research output for patient care [9]. The AS&HCN was established by the East of England Strategic Health Authority (SHA) to support the development of stroke services in three counties, Norfolk, Suffolk and Cambridgeshire. In the present report, we analysed the first month of ASCNESdata (October 2009). ASCNES consists of 4 retrospective (Oct 2009, Jan 2010, April 2010, July 2010) and 4 prospective (Oct 2010, Jan 2011, April 2011, July 2011) cohorts, each with a follow-up period of one year [9]. The AS&HCN, on the other hand, ceases the data collection at the point of patient’s discharge and continuously collects data of consecutive admissions in the same eight NHS Trusts in East of England.

For this study, the patients included were confirmed stroke cases (either ischaemic or haemorrhagic stroke) admitted to eight hospitals in the East of England region with the catchment population of ~2.5 million. Stroke was defined as a sudden onset of focal neurological deficit lasting greater than 24 hours as consequence of an intracerebral ischaemic or haemorrhagic event. All patients had cerebral imaging (CT or MRI) to confirm the diagnosis and patients with TIA were not included. Stroke cases were identified prospectively by the clinical team caring for the patient. Anonymised data from each hospital were sent on a monthly basis to the AS&HCN which collates the data on clinical service activities in order to evaluate the services in relation to National targets and guidance from the Royal College of Physicians [10].

ASCNES is funded by the NIHR Research for Patient Benefit Programme (PB-PG-1208-18240) and obtained ethical approval from the Norfolk Research Ethics Committee. The Anglia Stroke Clinical Network (part of AS&HCN) was funded by the NHS Improvement Programme through the East of England SHA. Through an additional data transfer agreement as part of the ASCNES, consecutive data was obtained from the AS&HCN for the period between September 2008 and April 2011. The main difference between the Oct 2009 data from ASCNES and AS&HCN data is that data completeness is better (i.e., less missing data) in ASCNES due to extra resources associated with the research project grant to collect data more fully from medical records.

### Data collection

Data collection was done by the clinical team at each participating hospital from routinely available clinical data. The variables used in the current study included the participants’ age, gender, pre-stroke residence status (categorical - home, care home, other), pre-stroke modified Rankin scale (mRS) [11], date of arrival, time of admission, presence of atrial fibrillation, type of stroke (ischaemic or hemorrhagic), Oxfordshire Community Stroke Project (OCSP) classification (lacunar stroke (LACS), partial anterior circulation stroke (PACS), total anterior circulation stroke (TACS), posterior circulation stroke (POCS)) [12], National Institute for Health Stroke Scale (NIHSS), systolic blood pressure, IV thrombolysis status, in-patient mortality and discharge destination. The pre-stroke mRs was assessed by specialist stroke nurses and doctors who scored pre-stroke disability according to pre-morbid mRS (0–5). When participants were admitted, stroke specialist nurses ascertained their premorbid (prestroke) mRs from nursing and medical records [13].

### Statistical analysis

The October 2009 data from ASCNES was compared to the AS&HCN (full cohort collected Sept 2008 to April 2011) data. The admissions in Oct 2009 were removed from the full cohort, as they are essentially the same patients as the Oct 2009 cohort of the ASCNES. In order to examine seasonality, the full cohort excluding patients admitted in Oct 2009 was broken down into three seasonal cohorts based on Curwen’s methods [14,15], where winter is defined as December to March in all years covered in the cohort, and the period in the preceding 4 months period is considered to be the Autumn cohort (August to November) and the following 4 months period is considered to be the Summer cohort (April to July).

Descriptive statistics were used to compare the characteristics of each cohort. Mean and standard deviation as well as median and inter quartile ranges for continuous variables and the number and percentages for categorical variables were reported. The t-test for continuous data and Pearson’s Chi^2^ test or Fisher’s exact test for categorical data were used.

The likelihood of in-patient mortality (Odds Ratio) was also examined, first unadjusted, and then adjusting for the selected variables which showed statistically significant differences in sample characteristics between Oct 2009 and other cohorts. Of the characteristics which were significantly different at 10% level in any inter-cohort comparison, age, gender and pre-stroke modified Rankin were chosen as covariates for adjustment. Pre-stroke modified Rankin was chosen especially for its data completeness as well as because our previous work indicates that it has an important independent effect on stroke outcome [13].

Month-to-month variations in mortality rates in stroke patients were examined graphically by comparing individual monthly rates (95%CI) to the mean (95%CI) of the whole population of the AS&HCN data.

## Results

We included a total of 8706 stroke patients which consisted of the October 2009 cohort (n = 308) from ASCNES and the full cohort (excluding October 2009, n = 8398) from the AS&HCN dataset. The full cohort was further broken down into the autumn cohort (August to November, excluding October 2009, n = 2184), winter cohort (December to March, n = 3478) and summer cohort (April to July, n = 2736).

The characteristics of the participants in each cohort are shown in Table 1. The mean age of the October 2009 cohort was higher than the remaining cohorts but the median age of all cohorts were similar. October 2009 had significantly more females than the autumn cohort, and borderline more than the full and winter cohorts. No statistically significant differences were observed for pre-stroke residence, pre-stroke modified Rankin score, weekend or weekday admission, time of admission, proportion of people with atrial fibrillation, type of stroke and use of thrombolysis for ischaemic stroke between cohorts.

**Table 1:** Comparison of stroke patients’ characteristics admitted between one month* and over 2 years** and during autumn, winter and summer months***to eight NHS Trusts in the East of England

**Variables**	**Oct 2009 cohort (n = 308)**	**Full cohort (n = 8398)**^**†**^		**Autumn cohort (n = 2184)**^**†**^		**Winter cohort (n = 3478)**^**†**^		**Summer cohort (n = 2736)**^**†**^	
		**Results**^**‡**^	**p-value**^**§ **^**vs. Oct 2009**	**Result**^**‡**^	**p-value**^**§ **^**vs. Oct 2009**	**Result**^**‡**^	**p-value**^**§ **^**vs. Oct 2009**	**Result**^**‡**^	**p-value**^**§ **^**vs. Oct 2009**
Age									
n (%)	258 (84)	8280 (99)		2162 (99)		3444 (99)		2674 (98)	
Mean (SD)	78.3 (11.9)	76.3 (13.0)	0.01^§^	76.5 (12.7)	0.03^§^	76.5 (12.8)	0.02^§^	75.9 (n = 2674)	< 0.01^§^
Median (IQR)	80 (72-87)	79 (70-86)	0.19^χ^	79 (70-86)	0.36^χ^	79 (69-86)	0.20^χ^	79 (69-85)	0.11^χ^
Gender			0.06^¥^		0.04^¥^		0.05^¥^		0.18^¥^
Female	172 (56)	4121 (51)		1050 (50)		1699 (50)		1372 (52)	
Male	134 (44)	3998 (49)		1057 (50)		1682 (50)		1259 (48)	
Pre-stroke residence			0.60^¶^		0.31^¶^		0.74^¶^		0.47^¶^
Home	276 (92)	5231 (90)		1533 (89)		2115 (90)		1583 (89)	
Care home	22 (7)	531 (9)		169 (10)		192 (8)		170 (10)	
Other	3 (1)	69 (1)		12 (1)		37 (2)		20 (1)	
Pre-stroke Rankin			0.07^¶^		0.08^¶^		0.05^¶^		0.13^¶^
0	128 (50)	2501 (52)		750 (51)		981 (51)		770 (53)	
1	54 (21)	813 (17)		245 (17)		342 (18)		226 (15)	
2	20 (8)	572 (12)		175 (12)		236 (12)		161 (11)	
3	28 (11)	545 (11)		167 (11)		210 (11)		168 (11)	
4	23 (9)	322 (7)		97 (7)		112 (6)		113 (8)	
5	2 (1)	103 (2)		33 (2)		42 (2)		28 (2)	
Weekend			0.36^¥^		0.59^¥^		0.31^¥^		0.32^¥^
Yes	84 (27)	2096 (25)		564 (26)		857 (25)		675 (25)	
No	224 (73)	6302 (75)		1620 (74)		2621 (75)		2061 (75)	
Time of admission			0.63^¥^		0.73^¥^		0.35^¥^		0.99^¥^
0900 to 1700	164 (53)	4045 (48)		1140 (52)		1755 (50)		1458 (53)	
1700 to 0900	144 (47)	4353 (52)		1044 (48)		1723 (50)		1278 (47)	
Prior atrial fibrillation			0.10^¥^		0.10^¥^		0.16^¥^		0.09^¥^
Yes	68 (35)	1123 (29)		347 (29)		407 (30)		369 (29)	
No	127 (65)	2710 (71)		850 (71)		952 (70)		908 (71)	
Type of stroke			0.23^¥^		0.06^¥^		0.36^¥^		0.43^¥^
Ischaemic	260 (89)	6533 (87)		1777 (85)		2657 (87)		2099 (88)	
Haemorrhagic	31 (11)	980 (13)		307 (15)		380 (13)		293 (12)	
OCSP classification			0.01^¥^		0.06^¥^		0.01^¥^		0.01^¥^
LACS	58 (23)	1195 (25)		360 (25)		480 (26)		355 (24)	
PACS	116 (47)	1815 (38)		566 (39)		694 (38)		555 (38)	
TACS	52 (21)	1012 (21)		324 (22)		373 (20)		315 (21)	
POCS	22 (9)	711 (15)		200 (14)		277 (15)		234 (16)	
NIHSS									
n (%)	46 (15)	1843 (22)	<0.01^§^	584 (27)	<0.01^§^	691 (20)	<0.01^§^	568 (21)	<0.01^§^
Mean (SD)	11.1 (7.3)	7.6 (6.6)	<0.01^χ^	7.7 (6.8)	<0.01^χ^	7.5 (6.4)	<0.01^χ^	7.6 (6.7)	<0.01^χ^
Median (IQR)	10 (5-16)	6 (2-12)		5 (2-12)		6 (3-12)		6 (2-12)	
Systolic BP									
n (%)	301 (98)	7021 (84)		1853 (85)		2951 (85)		2217 (81)	
Mean (SD)	155 (29)	158 (31)	0.09^§^	158 (31)	0.16^§^	159 (31)	0.05^§^	158 (30)	0.14^§^
Median (IQR)	152 (135-175)	155 (137-178)	0.10^χ^	155 (136-179)	0.13^χ^	157 (138-178)	0.03^χ^	154 (137-177)	0.54^χ^
Thrombolysis			0.10^¥^		0.08^¥^		0.08^¥^		0.17^¥^
Yes	5 (2)	294 (4)		128 (4)		128 (4)		85 (4)	
No	252 (98)	7008 (96)		2910 (96)		2910 (96)		2275 (96)	

*Abbreviations*: *SD* standard deviation, *IQR* interquartile range, *NIHSS* National Institute of Health Stroke Score, *OCSP* Oxfordshire Community Stroke Project, *LACS* lacunar stroke, *PACS* partial anterior circulation stroke, *TACS* total anterior circulation stroke, *POCS* posterior circulation stroke, *BP* blood pressure.

*October 2009.

**December 2008 to April 2011 excluding October 2009.

***August-Novembers 2009, and 2010 excluding October 2009.

^†^The October 2009 data was excluded.

^‡^Results reported as mean (standard deviation) and median (interquartile range) for continuous variables and number (%) for categorical data.

^§^T-test for continuous data. Statistical difference performed on complete data only.

^¥^Pearson Chi^2^ test for categorical data. Statistical difference performed on complete data only.

^¶^Fisher’s exact test for categorical data. Statistical difference performed on complete data only.

^χ^Pearson Chi^2^ test for comparison of median. Statistical difference performed on complete data only.

The OCSP classification of stroke showed some differences between cohorts as there were more PACS (47%) and less POCS (9%) in the October 2009 cohort compared to the other cohorts (PACS 38-39%, POCS 14-16%). However, there were similar rates of LACS and TACS. Statistically significant differences were observed for the National Institute of Health Stroke Score (NIHSS) between the October 2009 cohort and other cohorts but there was a high degree of missing data because the score was only calculated for patients potentially suitable for thrombolysis (< 30%).

**Figure 1:**
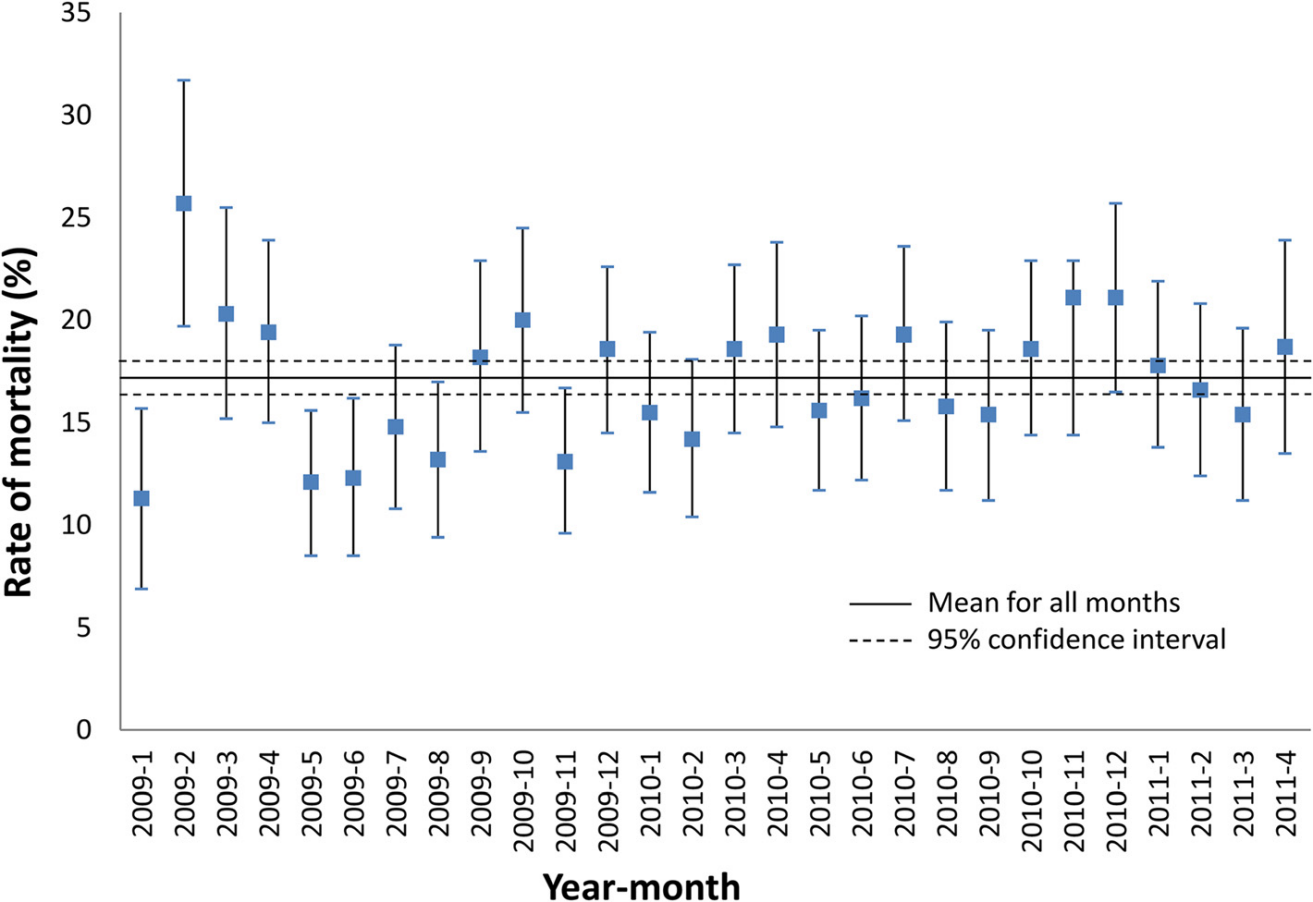
**Rate of in-patient stroke mortality (95% CI) across all months and for individual months from the AS&HCN dataset.** The October 2009 data is from AS&HCN dataset and not from the October 2009 cohort of ASCNES. Mortality is exactly the same as Oct 2009 in both datasets represents the same cohort.

The in-patient mortality and discharge destinations of the cohorts are shown in Table 2. The crude rates for in-patient mortality were not statistically different between the October 2009 cohort and the remaining cohorts. Examining the likelihood of in-patient mortality and discharge destination outcome by univariate and multiple logistic regression models did not show any significant difference.The monthly rates of in-patient mortality and overall in-patient mortality rate are shown in Figure 1. The mean mortality across all of the months was 17.2% (95% CI 16.4%-18.0%). Significantly higher rate of mortality was observed in February 2009 and significantly lower mortality rates were observed in January and May 2009 compared with the average rate. The in-patient mortality rate of the October 2009 cohort at 20% was higher than the seasonal averages. There was no consistent monthly pattern of in-patient deaths over the three calendar-years covered by the AS&HCN data.

**Table 2:** Comparison of stroke outcomes in patients admitted between one month* and over 2 years** and during autumn, winter and summer months*** admitted to the eight NHS Trusts in the East of England

**Variables**	**Oct 2009 cohort (n = 308)**	**Full cohort (n = 8398)**^**†**^		**Autumn cohort (n = 2184)**^**†**^		**Winter cohort (n = 3478)**^**†**^		**Summer cohort (n = 2736)**^**†**^	
		**Results**^**‡**^	**p-value**^**§ **^**vs. Oct 2009**	**Result**^**‡**^	**p-value**^**§ **^**vs. Oct 2009**	**Result**^**‡**^	**p-value**^**§ **^**vs. Oct 2009**	**Result**^**‡**^	**p-value**^**§ **^**vs. Oct 2009**
Death			0.13		0.10		0.25		0.09
Yes	62 (20)	1414 (17)		357 (16)		609 (18)		448 (16)	
No	246 (80)	6984 (83)		1827 (84)		2869 (82)		2288 (84)	
Likelihood of death Unadjusted OR	1.00	0.80 (0.60-1.07) N = 8706	0.13	0.78 (0.57-1.04) N = 2492	0.10	0.84 (0.63-1.13) N = 3786	0.25	0.78 (0.58-1.04) N = 3044	0.10
Adjusted OR^¥^	1.00	0.85 (0.60-1.22) N = 4877	0.39	0.88 (0.60-1.28) N = 1613	0.49	0.93 (0.64-1.34) N = 2078	0.70	0.76 (0.52-1.12) N = 1620	0.17
Discharge residence			0.21						
Home	165 (71)	4115 (67)		1082 (66)	0.19	1701 (69)	0.33	1332 (66)	0.13
Care home	24 (10)	884 (14)		238 (15)		341 (14)		305 (15)	
Other	44 (19)	1133 (18)		318 (19)		440 (18)		375 (19)	

*October 2009.

**December 2008 to April 2011 excluding October 2009.

***August-Novembers 2009, and 2010 excluding October 2009.

^†^The October 2009 data was excluded.

^‡^Results reported as mean (standard deviation) and median (interquartile range) for continuous variables and number (%) for categorical data.

^§^Chi^2^ test for categorical data. Statistical difference performed on complete data only.

^¥^Logistic regression adjusted for age, gender and pre-stroke Rankin. Odds ratios and corresponding 95% confidence intervals are reported.

## Discussion

Our results suggest that, in this stroke patient population in East of England, one month consecutive data with ~300 participants is similar in most respects in composition and discharge outcome to data from over two years with > 8000 stroke patients. Furthermore, there were few significant differences in baseline characteristics and discharge destination and inpatient mortality of stroke patients between one-month data and three seasonal averages over 2 years. This suggests that some patient characteristics and outcomes may be representative of those admitted over longer periods when clinical evaluations are as short as one month in duration, provided an adequate number of consecutive admissions are considered.

However, we found differences in stroke classification and NIHSS score between one month data and data collected over 2 years. There was a significant amount of missing data for NIHSS scores and we believe that collection of NIHSS data may be biased towards patients who were potential candidates for thrombolysis treatment. Differences observed in OCSP classification may indicate that one month data may not adequately represent case-mix.

The data collection period is important in methodological considerations of service evaluation and epidemiological research. Large sample sizes usually allow adequate study power. Case-mix is also important because it can influence outcomes, particularly in non-randomised studies [16]. We previously analysed over five thousand stroke admissions over six years and found a winter excess in hospital stroke admissions, death and length of acute hospital stay [17]. However, there is some conflicting evidence to suggest that there is no seasonal effect [18]. We have shown that in this data set there is little different in some baseline variables for stroke admissions during one month data collection compared to data collected over two years. This provides evidence that one month data may adequately capture variations in case-mix and seasonal effects.

Clinical network registry data are valuable in audit, health service evaluation and offer several advantages compared to other methods of data collection. Compared to retrospective data collection, the data from networks may be prospectively collected, either linked directly from medical records or collected as patients are admitted. As networks, data collection across multiple hospitals not only increases the sample size but also addresses inter-hospital variations. Prospective identification of cases and standardised data collection allow for consistency in information extracted. The observational nature of networks lack strict inclusion and exclusion criteria such as those in the clinical trials and includes all cases that meet the network definition, which may permit generalisation to a population of interest.

We made several comparisons by breaking down the full cohort into three separate seasonal cohorts to search for evidence of anyaveraging effect. This is because the representativeness of one month compared to 2 years may be a by chance observation and may not capture variations that occur over 2 years. However, there was no evidence to suggest this was the case as Oct 2009 was similar to three different seasonal cohorts over 2 years. ASCNES [9] will collect eight selected months’ data (every 3rd month over 2 years) and therefore our study methodology is likely to be very robust. Future observational studies in evaluation of stroke services can learn from this experiment and will be able to design their studies in a cost efficient way as exemplified in ASCNES.

Month-to-month variation in stroke outcomes and severity may be related to seasonal differences. Studies have suggested that seasonal observations in stroke outcomes and severity may relate to changes in outdoor temperature and related meteorological parameters [19]. Our results however do not support the presence of a consistent seasonal effect on patient characteristics admitted to the stroke units as well as mortality. Aside from January, February and May 2009, all other months showed no significant difference to the overall mean monthly mortality rate. However, a caveat is that the fact that the seasonal data were over 2–3 years and this might have dampened seasonal effects in any particular year – as they would be averaged over the other years. One plausible explanation for this is that there is no seasonal difference in the behaviour of stroke patients in terms of hospital use and the findings of other studies may simply reflect expected variations of health care utilisation in different patient populations.

This study has several strengths. This study has a large sample size. We included eight diverse NHS trusts thus allowing us to capture variation in the case-mix and the outcomes are generalisable to the UK NHS setting. We used routinely collected administrative data to identify participants and a recent pilot validation study found this approach is reliable [20].

This study has some limitations. Variables such as presence of atrial fibrillation, NIHSS and use of thrombolysis had a high number of missing data. We observed a high amount of missing NIHSS dataand believe that this data collection may be biased because the NIHSS score may not have been collected if the patient was not suitable for thrombolysis. Furthermore, it could be argued that there may be a degree of inter-observer variability in collection of pre-stroke disability and NIHSS scores. However random measurement error will only attenuate the associations. We were not able to control for patient co-morbidities but we have adjusted for pre-stroke mRs which is indicative of physical functioning contributed by major co-morbidities. The study population is relatively homogenous (white Caucasians) and also is confined to one UK region which perhaps has less extreme weather conditions. It is possible that the results are due to chance but we have compared the objective hard outcome of mortality rate across all months to observe if there were differences.

## Conclusions

Our results suggest that, in this cohort, data from one month is similar to data collected over two years and to seasonal averages over two to three years. This suggests that it may be valid to conduct clinical evaluations as short as one month in duration with a low risk of encountering problems with a case-mix and seasonality provided the study sample size is appropriately powered for the outcome/s of interest. Although we have shown that similarity between one month and longer term data collection, until further accumulating body evidence suggests similarly in different populations and settings, studies of longer duration are recommended because these studies are more likely to capture the variations in case-mix more comprehensively.

### Ethics

Ethical approval was obtained from the Norfolk Research Ethics Committee and Institutional Approvals were obtained for the use of AS&HCN data.

## Abbreviations

ASCNES: Anglia stroke clinical network evaluation study; AS&HCN: Anglia stroke& heart clinical network; rTPA: Thrombolysis; SHA: Strategic health authority; mRS: Modified rankin scale; OCSP: Oxfordshire community stroke project; LACS: Lacunar stroke; PACS: Partial anterior circulation stroke; TACS: Total anterior circulation stroke; POCS: Posterior circulation stroke; NIHSS: National institute for health stroke scale.

## Competing interests

The authors declare that they have no competing interests.

## Authors’ contributions

PKM conceptualize the study. GD is the director of the Anglia Stroke and Heart Network who oversaw the data collection. CSK, SDM and GMP performed the data analysis. CSK and PKM wrote the first draft of the manuscript and all of the authors contributed to the manuscript and approved the final manuscript.
